# Impact of Acute High Glucose on Mitochondrial Function in a Model of Endothelial Cells: Role of PDGF-C

**DOI:** 10.3390/ijms24054394

**Published:** 2023-02-23

**Authors:** Adriana Grismaldo Rodríguez, Jairo Zamudio Rodríguez, Alfonso Barreto, Sandra Sanabria-Barrera, José Iglesias, Ludis Morales

**Affiliations:** 1 Experimental and Computational Biochemistry Group, Faculty of Sciences, Nutrition and Biochemistry Department, Pontificia Universidad Javeriana, Bogotá 110231, Colombia; 2 Immunology and Cell Biology Group, Faculty of Sciences, Microbiology Department, Pontificia Universidad Javeriana, Bogotá 110231, Colombia; 3 Bioengineering FCV Research Group, Department of Innovation and Technological Development, Fundación Cardiovascular de Colombia, Floridablanca 680004, Colombia

**Keywords:** endothelial cells, high glucose, PDGF-C, mitochondrial dynamics, bioenergetics

## Abstract

An increase in plasma high glucose promotes endothelial dysfunction mainly through increasing mitochondrial ROS production. High glucose ROS—induced has been implicated in the fragmentation of the mitochondrial network, mainly by an unbalance expression of mitochondrial fusion and fission proteins. Mitochondrial dynamics alterations affect cellular bioenergetics. Here, we assessed the effect of PDGF-C on mitochondrial dynamics and glycolytic and mitochondrial metabolism in a model of endothelial dysfunction induced by high glucose. High glucose induced a fragmented mitochondrial phenotype associated with the reduced expression of OPA1 protein, high DRP1^pSer616^ levels and reduced basal respiration, maximal respiration, spare respiratory capacity, non-mitochondrial oxygen consumption and ATP production, regarding normal glucose. In these conditions, PDGF-C significantly increased the expression of OPA1 fusion protein, diminished DRP1^pSer616^ levels and restored the mitochondrial network. On mitochondrial function, PDGF-C increased the non-mitochondrial oxygen consumption diminished by high glucose conditions. These results suggest that PDGF-C modulates the damage induced by HG on the mitochondrial network and morphology of human aortic endothelial cells; additionally, it compensates for the alteration in the energetic phenotype induced by HG.

## 1. Introduction

Metabolic diseases, including diabetes, are considered the main risk factor for the development of cardiovascular diseases (CVD) [1,2]. The origin of CVD has been related to early loss of vascular endothelial function, which decreases the production or bioavailability of vasodilator molecules such as nitric oxide (NO) and predisposes to a blood vessel contraction phenotype [3]. Transient and sustained glucose levels greater than 5.5 mmol/L considered average fasting glucose levels [4] induce endothelial dysfunction [5,6,7]. Consequently, identification of the initial steps that lead to endothelial dysfunction under high glucose conditions is crucial for early intervention in diabetes.

Reactive oxygen species (ROS) production, especially superoxide radical (O^−^) by the mitochondria, is one of the intracellular mechanisms that reduce the biodisponibility of NO. Due to the high reactivity of O^−^ and NO, the production of peroxynitrite (ONOO^-^) is promoted, altering the structure of nucleic acids, proteins, and lipids and leading to cell death [8]. Although the mitochondrial content in endothelial cells (ECs) is low compared to other cell types with higher energy demands [9], they are considered signalling organelles that act as local microenvironmental sensors [9,10,11]. Its optimal function is driven by a balance between fission and fusion processes [3,12,13,14,15]. However, in diabetic patients and hyperglycemic conditions, a decreased expression of OPA1 and MFN1/2 proteins (related to the fusion mechanism) and an increased expression of DRP1 and FIS1 proteins (related to the fission mechanisms) are observed. This imbalance, coupled with an impaired autophagy mechanism, leads to a disrupted mitochondrial network and the accumulation of tiny, damaged, and inefficient organelles that contribute to the increased ROS production and loss of ECs function or death [12,13,14,16].

In this context, looking for new therapies that mitigate the mitochondrial damage induced by high glucose is critical for the reduction of CVD risk in diabetic patients.

Recently, we reported the role of PDGF-C on the modulation of mitochondrial oxidative stress induced by high d-glucose in human aortic endothelial cells (HAECs). PDGF-C is a growth factor that exerts its effects by binding to PDGFRα and PDGFR αβ tyrosine kinase receptors. PDGF-C reduced the increase in mitochondrial superoxide production, and it was associated with the up-regulation of SOD2 expression and activity and the modulation of *Keap1* gene expression [5]. Now, here we report the effect of this growth factor on the modulation of the fragmented mitochondrial morphology and the mitochondrial functional changes induced by high glucose in endothelial cells.

## 2. Results

### 2.1. PDGF-C Restores the Integrity of Mitochondrial Network of HAECs Going on High d-Glucose Treatment

**Figure 1:**
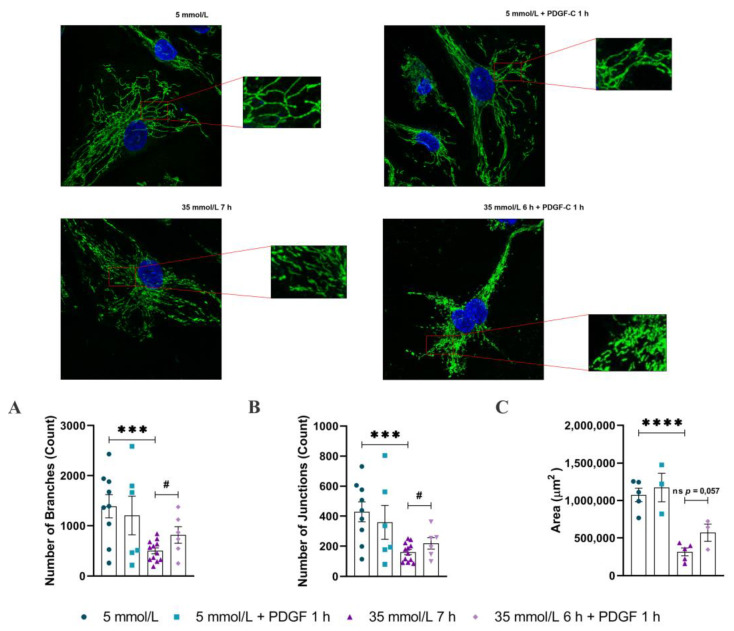
Effect of PDGF-C on mitochondrial morphology changes induced by high d-glucose. HAECs were seeded in 35 mm glass-bottom culture dishes and exposed to 5 or 35 mmol/L d-glucose for 7 h, treated or not with 50 ng/mL PDGF-C for the last hour of the glucose exposure. Live cells were stained with Mitotracker green (mitochondria visualization) and Hoechst (nucleus visualization) before the confocal images were acquired for analysis of mitochondrial network integrity. Representative images for cells in NG (upper left), NG+PDGF-C (upper right), HG (lower left) and HG+PDGF-C (lower right). Analysis of mitochondrial network integrity represented by the count of (**A**) branches (n NG: 9, n NG+PDGF: 6, n HG: 12, n HG+PDGF: 6), (**B**) count of junctions (n NG: 9, n NG+PDGF: 6, n HG: 12, n HG+PDGF: 6), and (**C**) mitochondrial area (n NG: 5, n NG+PDGF: 3, n HG: 5, n HG+PDGF: 3). Data represent the mean ± SEM of three independent experiments. *** *p* < 0.001 and **** *p* < 0.0001 regarding NG, # *p* < 0.05 regarding HG, ns (non-significant).

To better understand the effect of PDGF-C on mitochondrial damage induced by 35 mmol/L of d-glucose for 7 h (herein referred to as HG) in HAECs, mitochondrial network integrity was evaluated by confocal microscopy. As shown in Figure 1A, mitochondria of cells cultured in 5 mmol/L of d-glucose (herein referred to as NG) exhibited a continuous and elongated network with peripheral localization (upper left and right). In contrast, HG induced a shorter and fragmented mitochondrial morphology (lower left), which changes to dense and hyperfused aggregates with nuclear localization when cells were treated with 50 ng/mL of PDGF-C for 1 h (lower right). The reduction of 64% of the count of branches (*** *p* < 0.001) (Figure 1A), 63% of the count of junctions (*** *p* < 0.001) (Figure 1B), and 71% of the mitochondrial area (**** *p* < 0.0001) (Figure 1C)) in HG conditions for 7 h and compared to NG support the morphology observations. Treatment with PDGF-C 50 ng/mL for 1 h significantly increased the number of branches (# *p* < 0.05), junctions (# *p* < 0.05) and a tendency to increase the total mitochondria area of cells treated with HG. These results suggest that PDGF-C modulates the damage induced by HG on the mitochondrial network and morphology of HAECs.

### 2.2. Mitochondrial Dynamic-Related Proteins Expression

**Figure 2:**
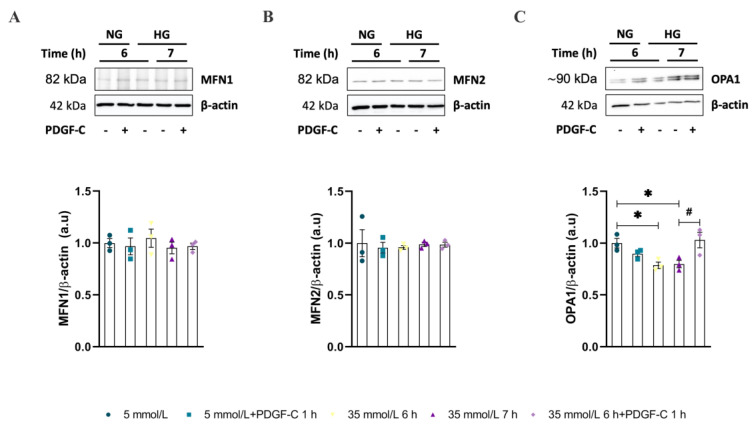
Effect of PDGF-C on fusion-related proteins in HAECs exposed to high glucose. Cells were seeded in 6 well plates and exposed to HG (35 mmol/L d-glucose) for 6 and 7 h, treated or not with 50 ng/mL PDGF-C for 1 h, and (**A**) MFN1, (**B**) MFN2, and (**C**) OPA1 expression was evaluated by western blot. Images correspond to representative blots for each protein. Densitometry analysis corresponds to the band of each protein normalized with the band of β-actin. Data represent the mean ± SEM of at least three independent experiments (* *p* < 0.05, # *p* < 0.05 regarding HG).

To reinforce these results, the expression of fission and fusion proteins was measured by western blot in the same conditions mentioned above. Regarding NG conditions, and as described in Figure 2A,B (Supplementary Figure S1A,B), HG did not significantly change MFN1 and MFN2 expression. Similarly, PDGF-C did not affect the expression of these fusion proteins in any of the evaluated glucose conditions and times. Opposite, HAECs going on HG for 6 and 7 h diminished the OPA1 protein expression (Figure 2C, Supplementary Figure S1C), regarding NG (* *p* < 0.05); PDGF-C treatment restored to the basal level, the expression of this fusion-related protein (# *p* = 0.0486).

**Figure 3:**
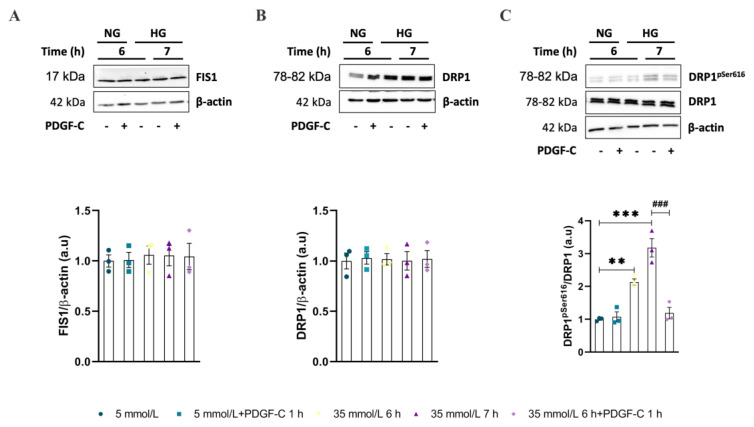
Effect of PDGF-C on fission-related proteins in HAECs exposed to high glucose. Cells were seeded in 6 well plates and exposed to 35 mmol/L d-glucose for 6 and 7 h without and with 50 ng/mL PDGF-C for 1 h, and (**A**) FIS1 and (**B**) DRP1 expression and the ratio between (**C**) DRP1^pSer616^/DRP1 were evaluated by western blot. Images correspond to representative blots for each protein. Densitometry analysis corresponds to the band of each protein normalized with the band of β-actin and DRP1 for phosphorylation residue. Data represent the mean ± SEM of at least three independent experiments. (** *p* < 0.01, *** *p* < 0.001, ### *p* < 0.05 regarding HG).

On the other hand, results about mitochondrial fission-related proteins showed that HG did not change the expression of either FIS1 or DRP1 alone or in combination with PDGF-C (Figure 3A,B, respectively. Supplementary Figure S2A,B). Additionally, the phosphorylation of DRP1 at Ser616, which is known for promoting the fission of the mitochondrial network [17,18], also was evaluated by western blot. As shown in Figure 3C (Supplementary Figure S2C), HG for 6 (** *p* < 0.01) and 7 h (*** *p* < 0.001) increased the phosphorylation of Ser616 residue in DRP1 and PDGF-C treatment diminished this to basal level (### *p* < 0.001).

These results suggest that PDGF-C modulates the mitochondrial network and morphology by regulating the fission through phosphorylation and dephosphorylation of DRP1 and intensifying the fusion process by upregulating OPA1 expression in HAECs going on HG conditions.

### 2.3. Bioenergetic Analysis

**Figure 4:**
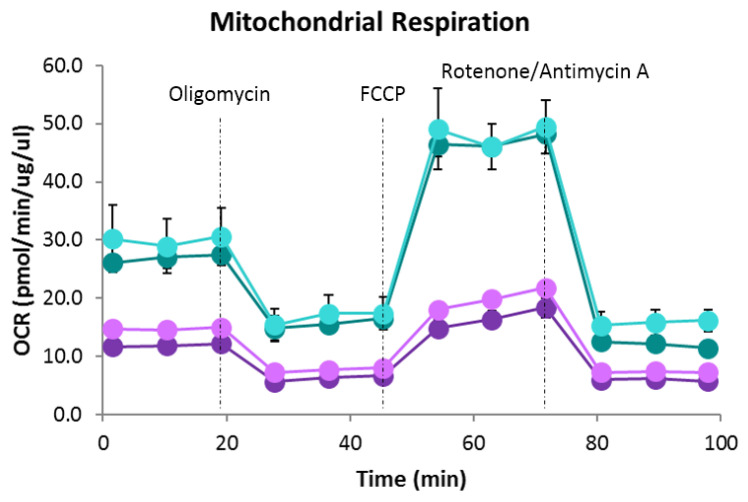

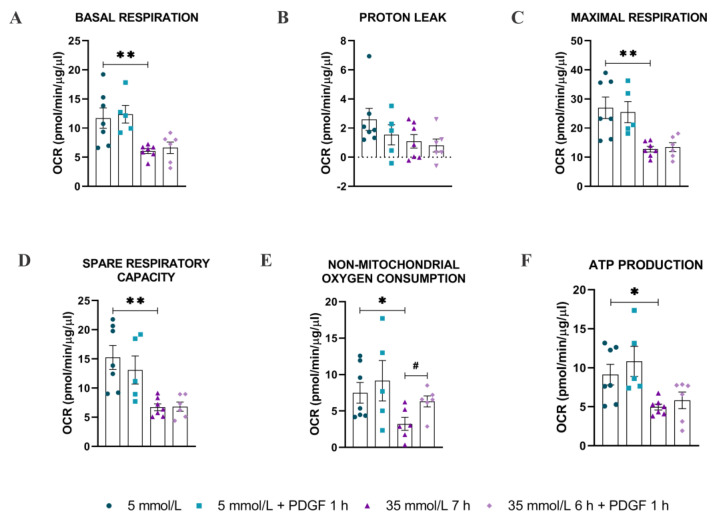
Effect of HG and PDGF-C on mitochondrial respiration of HAECs. Cells were seeded in 24 well plates for Seahorse and exposed to 35 mmol/L d-glucose for 7 h without and with 50 ng/mL PDGF-C for the last hour, and mitochondrial respiration was estimated (central upper panel: dark green: 5 mmol/L, light green: 5 mmol/L + PDGF-C 1 h, dark purple: 35 mmol/L for 7 h and light purple: 35 mmol/L + PDGF-C 1 h). (**A**) Basal respiration, (**B**) Proton leak, (**C**) Maximal respiration, (**D**) Spare respiratory capacity, (**E**) Non-mitochondrial oxygen consumption and (**F**) ATP production are shown as the mean ± SEM of two independent experiments with multiple replicates (** *p* < 0.01, * *p* < 0.05 and # *p* < 0.05 regarding HG).

To know the implications of acute elevated high glucose concentrations on the mitochondrial function of HAECs and the role of PDGF-C in these conditions, cells were treated as mentioned above. The oxygen consumption rates (OCRs) were measured with Agilent Seahorse XFe24 Analyzer Mitostress Test (Seahorse Bioscience, Agilent, Santa Clara, CA, USA), according to the manufacturer’s protocol [16,19]. The live-cell bioenergetics was conducted to determine the basal mitochondrial functions, including oxygen consumption rates (OCR), extracellular acidification rates (ECAR), ATP production, proton leak, maximal respiration, spare respiratory capacity, mitochondrial stress, and nonmitochondrial respiration. Basal OCR and OCR in response to injection of oligomycin (ATP synthase inhibitor), FCCP (mitochondrial uncoupler), and rotenone/antimycin (Complex I and III inhibitors, respectively; Figure 4, central upper panel) were assayed. Evaluation of the six mitochondrial parameters showed that HG significantly reduced basal respiration (** *p* < 0.01; Figure 4A), maximal respiration (** *p* < 0.01; Figure 4C), spare respiratory capacity (**p < 0.01; Figure 4D), non-mitochondrial oxygen consumption (* *p* < 0.05; Figure 4E) and ATP production (* *p* < 0.05; Figure 4F), regarding NG conditions. PDGF-C significantly increased the non-mitochondrial oxygen consumption diminished by HG conditions (# *p* < 0.05; Figure 4E).

**Figure 5:**
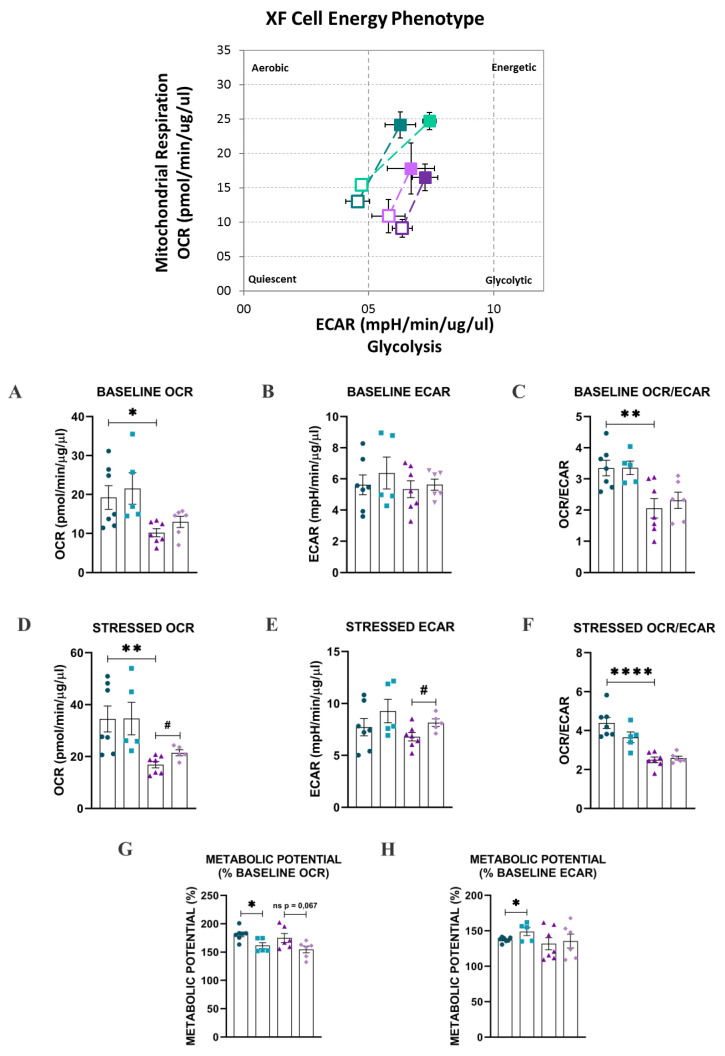
Effect of HG and PDGF-C on energy phenotype of HAECs. Cells were seeded in 24 well plates for Seahorse, exposed to 35 mmol/L d-glucose for 7 h without and with 50 ng/mL PDGF-C for the last hour, and energy phenotype was estimated (central upper panel. Empty squares: baseline, filled squares: stressed. Dark green: 5 mmol/L, light green: 5 mmol/L + PDGF-C 1 h, dark purple: 35 mmol/L for 7 h and light purple: 35 mmol/L + PDGF-C 1 h). (**A**) Baseline OCR, (**B**) Baseline ECAR, (**C**) Baseline OCR/ECAR ratio, (**D**) Stressed OCR, (**E**) Stressed ECAR, (**F**) Baseline OCR/ECAR ratio, (**G**) Metabolic potential (% OCR), and (**H**) Metabolic potential (% ECAR) are shown as the mean ± SEM of two independent experiments with multiple replicates (**** *p* < 0.0001, ** *p* < 0.01, * *p* < 0.05, and # *p* < 0.05 regarding HG).

In the same experiments, the parameters baseline phenotype, stressed phenotype (after oligomycin injection), and metabolic potential were evaluated to assess the cell energy metabolism phenotype (Figure 5, central upper panel) of HAECs going on NG and HG conditions, and the effect of PDGF-C on changes induced by HG. As shown in Figure 5, HG significantly reduced the baseline OCR (* *p* < 0.05; Figure 5A), the baseline OCR/ECAR ratio (** *p* < 0.01; Figure 5C), the stressed OCR (** *p* < 0.01; Figure 5D) and the stressed OCR/ECAR ratio (**** *p* < 0.0001). PDGF-C increased the stressed OCR (* *p* < 0.05; Figure 5D) and stressed ECAR (* *p* < 0.05; Figure 5E) and slightly reduced the metabolic potential (% baseline OCR; non-significant; Figure 5G). Interestingly, PDGF-C significantly reduced the metabolic potential (% baseline OCR; # *p* < 0.05; Figure 5G) and increased the metabolic potential (% baseline ECAR; # *p* < 0.05; Figure 5H) in basal d-glucose conditions (5 mmol/L), suggesting that PDGF-C potentiates the glycolytic metabolism even in normal glucose conditions.

## 3. Discussion

Although mitochondrial content in endothelial cells is low because of their low energy demand [9] and their mainly glycolytic ATP production [7,20,21,22,23], they have a long and extensive mitochondrial network that undergoes balanced cycles of fission and fusion and exerts essential functions related with environmental sensing and signaling [9,10,11], and maintaining the balance among calcium concentrations, ROS production and nitric oxide [23].

Mitochondrial network fragmentation has been previously reported in endothelial cells and in in vivo models of high glucose environment and diabetes [3,12,13,14,15]; this condition has been associated with the development of vascular dysfunction [3,15]. It is clearly stated the influence of increased ROS production in the induction of mitochondrial fission [24,25]; our results support these affirmations. In a previous study published by our group, we found augmented mitochondrial ROS in HAECs treated with HG for 6 to 9 h. It was related to the diminished expression of the antioxidant enzyme SOD2 and the activity of the Nrf2/Keap1 pathway [5]. Now here, in the same endothelial model, we report the induction of mitochondrial network fragmentation by HG conditions, reflected as short and discontinuous mitochondria localized at the cellular periphery, reduction in the count of branches, junctions, and total area, diminished expression of fusion protein OPA1, and augmented levels of DRP1^pSer616^, regarding NG conditions (Figure 1 and Figure 2). Concerning the PDGF-C effect, there are no reports about its involvement in the mitochondrial dynamics process associated with any pathology, so this is the first report showing PDGF-C as a mitochondrial morphology modulator in endothelial cells subjected to metabolic stress conditions. The mechanisms could be associated with the induction of SOD2 expression and consequent reduction in mitochondrial ROS production [5], which could regulate the mitochondrial fission mechanisms and maintain mitochondrial integrity and functionality [26].

Although no changes in DRP1 expression were observed, it is known that its pro-fission role depends on its translocation from the cytoplasm to the mitochondrial outer membrane [24]. This process is controlled by the phosphorylation of Ser616 and Ser637 residues [24,27]. In our model, HG conditions induced the phosphorylation of DRP1 in Ser616, which promotes the transit of DRP1 to mitochondria and leads to its fragmentation [17]; interestingly, this effect was reverted by PDGF-C treatment, possibly through the parallel activation of phosphatases whose target is DRP1; however, the main mechanisms remain unclear. In this context, the increased expression of OPA1 and the modulation of phosphorylation of DRP1 in Ser616 residue induced by PDGF-C probably promotes the fusion of dysfunctional mitochondria, leading to a distribution of damaged components, including mitochondrial DNA, uncoupling proteins, and antioxidant enzymes [13,14]. Additionally, the phosphorylation of DRP1 on Ser637 residue is known for reversing the effects of Ser616 phosphorylation [24]. Although the phosphorylation state of this residue was not evaluated in our study, it is known that PDGF-C drives different signalling pathways, including PI3K/Akt, MAPK, and PLCγ [28], leading to the activation of kinases such as AMPK, MAPK, and cyclin-dependent kinase 1/cyclin B1, involved in the phosphorylation of this residue [24].

Changes in mitochondrial morphology induced by environmental conditions, such as increased extracellular glucose levels, can alter the typical mitochondrial bioenergetics profile [29]. As shown in our results, HG-induced alterations in mitochondrial function are evidenced by diminishing basal respiration, maximal respiration, reserve capacity, non-mitochondrial OCR, and ATP-linked OCR (Figure 4). The reduction in maximal respiration, reserve capacity, and ATP-linked OCR has been related to diminished mitochondrial mass, mitochondrial dysfunction, low ATP demand and severe electron transport chain damage, respectively [16,19]; which is according with the diminished total mitochondrial area, mitochondrial network fragmentation (fewer branches and junctions regarding NG conditions) observed by confocal microscopy (Figure 1) and the reduction of mitochondrial fusion evidenced by the diminished expression of OPA1 (Figure 2). Although the non-mitochondrial OCR has been related to the increased production of extramitochondrial ROS (cytosolic) [16,19], in our model, we did not find evidence that suggests the high production of cytosolic ROS in EC exposed to HG [5]. Our results are supported by different studies that indicate that DRP1-induced mitochondrial fission is associated with a diminished OXPHOS capacity and the increased activity of glycolytic metabolism [24,30]. On these affected parameters, PDGF-C recovered the mitochondrial morphology, possibly through increasing the expression of the mitochondrial fusion protein OPA1; however, PDGF-C only exerted a restauration role on the non-mitochondrial OCR parameter (Figure 4E), which could be related to the induction of the initial response of endothelial cells to metabolic stress.

Even though our results indicate a diminished OXPHOS activity in HG cells (Figure 4 and Figure 5), proteomic analysis by [31] shows that energy production in diabetic primary rat cardiac microvascular endothelial cells (RCMVECs) is shifted from glycolysis to OXPHOS after high glucose stress (25 mM by 2 weeks). However, we demonstrated that acute HG stress (7 h) in non-diabetic human aortic endothelial cells decreases OXPHOS metabolism (Figure 4) by the assessment of oxygen consumption and, similarly, Hapsula et al., 2019 [31], reported diminished oxidative phosphorylation and increased glycolysis-related protein expression in non-diabetic RCMVECs after HG exposure, regarding cells in NG conditions. These results suggest differential metabolic responses to HG exposure dependent on cell origin and phenotype (i.e., microvasculature vs microvasculature, diabetic vs non-diabetic).

Typically, when extracellular glucose increases, the endothelial cells enhance the glucose uptake mainly through GLUT 1 transporters and metabolism through glycolysis and glycolytic side branches [32], while the OXPHOS capacity diminishes, as reported by [23] in the EA.hy926 cell line and confirmed by our results (Figure 4). Similarly, in a high glucose HUVECs model, Zeng et al., 2019 [33] evidenced an unbalanced process of mitochondrial dynamics promoting fission through the decreased expression of MFN1 and increased expression of FIS1, which was associated with the decreased expression of complex I (NADH: ubiquinone oxidoreductase core subunit 1) and complex II (Succinate dehydrogenase) of the electron transport chain, leading to a deficient aerobic metabolism.

Our results suggest that PDGF-C modulates the damage induced by HG on the mitochondrial network and morphology; additionally, it compensates for the alteration in the energetic phenotype induced by HG. Nevertheless, our work proposes an initial approach to show the changes that acute HG induces in a macrovascular endothelial cell model and the role that PDGF-C can exert on these changes. It constitutes a guide for future experiments, including the assessment of endothelial function parameters (i.e., angiogenic capacity, nitric oxide production) and the evaluation of the behavior of each mitochondrial complex in the established conditions.

## 4. Materials and Methods

### 4.1. Cells and Reagents

Human Aortic Endothelial Cells (CC-2535), EGM-2 BulletKit (CC-3162) and EBM-2 (00190860) were obtained from Lonza (Walkersville, MD, USA). hrPDGF-C (SRP3139) and Valinomycin (V0627) were obtained from Sigma-Aldrich (St. Louis, MO, USA). Mitotracker Green FM (M7514), Hoechst 34580 (H21486) N,N-dimethyl-4-[5-(4-methyl-1-piperazinyl)[2,5′-bi-1H-benzimidazol]-2′yl] and SuperSignal^TM^ west pico PLUS chemiluminescent substrate (34577) were obtained from Thermo Fisher Scientific/Invitrogen (Chelmsford, MA, USA). Protease and phosphatase inhibitor cocktail (5872), antibodies against OPA1 (D7C1A), MFN1 (D6E2S), MFN2 (D1E9), DRP1 (D6C7), DRP1^pS616^, β actin (8H10D10), Anti-rabbit IgG HRP-linked and anti-mouse IgG HRP-linked were obtained from Cell Signaling Technology (Danvers, MA, USA). Antibody against (ab96764) were obtained from Abcam (San Francisco, CA, USA).

### 4.2. Cell Culture and Treatments

All experiments were established according to the conditions selected before and reported in [5]. Briefly, Human Aortic Endothelial Cells (HAECs) from passage 4 to passage 7 were grown in standard culture conditions in EGM-2 BulletKit containing 5.5 mmol/L glucose (normal human fasting blood sugar average) [4]. Confluent cells were seeded in multiwell plates, and after 24 h, cells were deprived in an EBM-2 medium containing 5.5 mmol/L glucose and 0.2% fetal bovine serum. After 12 h, cells were treated with 29.5 mmol/L d-glucose to reach a final concentration of 35 mmol/L (HG) for 6–9 h; these times were selected according to a previous study where we identified increased production of mitochondrial ROS after 6–9 h of HG [5]. Treatments with 50 ng/mL of hrPDGF-C were made for 1 h after 6 h of 35 mmol/L d-glucose stress induction, considering the short half-life of PDGF in HUVECs, which has been reported to be between 50 min and 3 h [34]. All comparisons were made from cells treated with glucose 5.5 mmol/L.

### 4.3. Mitochondrial Network Analysis

HAECs were seeded at 3 × 10^4^ cells/well in 35 mm glass-bottom culture dishes (MatTek) coated with 0.2% gelatin and cultured until 60% of confluence. Once deprived for 12 h, cells were treated with d-glucose 35 mmol/L for 6 h and 1 additional hour with 50 ng/mL of PDGF-C to evaluate mitochondrial network integrity. Briefly, live cells were washed once with PBS and stained with 100 nmol/L Mitotracker Green FM and 5 μg/mL Hoechst to define mitochondria and nucleus, respectively [35]. 2D and 3D cell imaging were acquired with an Olympus FV1000 confocal microscope, using a 60× oil immersion objective and an excitation/emission range of 400/545 for MitoTracker Green FM and 361/497 for Hoechst. Images were pre-processed according to the protocol suggested by Chaudhry et al., 2020 [36], data about the count and length of branches, count of junctions, and total mitochondria area were obtained according to the protocol suggested by Valente et al., 2017 [37], using the Fiji plugin for Image J.

### 4.4. Mitochondrial Dynamics-Related Proteins Expression

Expression of mitochondrial fusion OPA1, MFN1, MFN2 and fission DRP1, and FIS1 proteins was measured by western blot. HAECs were seeded in 6-well plates, treated as above, and lysed on ice in RIPA buffer supplemented with protease and phosphatase inhibitors cocktail. Total protein was quantified by bicinchoninic acid. Obtained protein was electrophoresed and transferred to PVDF membranes. Membranes were incubated overnight at 4 °C with the antibodies and dilutions mentioned in Table 1. The next day, membranes were washed and incubated with anti-rabbit IgG HRP-linked or anti-mouse IgG HRP-linked (Table 1) antibodies at room temperature for 1 h. Protein bands were detected with SuperSignal^TM^ west pico PLUS chemiluminescent substrate and captured by the iBright 1500 imaging system from ThermoFisher Scientific (Chelmsford, MA, USA). Analysis of obtained bands was evaluated by densitometry with Image J software [38].

### 4.5. Bioenergetics Analysis

Oxygen consumption rate (OCR) and Extracellular acidification rate (ECAR) were measured in a Seahorse XFe24 analyzer (Seahorse Biosciences, MA, USA) through mito stress and energy phenotype tests, respectively. Cells were plated in a Seahorse microplate at a density of 7 × 10^4^ cells/well and treated as mentioned before. After completing the above-mentioned treatments, cells were equilibrated in DMEM without sodium bicarbonate, containing 5 mmol/L or 35 mmol/L (according to cell treatments) of d-glucose, 2 mmol/L of glutamine and 1 mmol/L of sodium pyruvate. Basal OCR and OCR in response to sequential injection of Oligomycin 1.5 μmol/L (ATP synthase inhibitor, mitochondrial complex V), FCCP 1 μmol/L (mitochondrial uncoupler), and Rotenone/Antimycin 0.5 μmol/L (mitochondrial complexes I and III inhibitors, respectively) were registered. Parameters such as proton leak, maximal respiration, spare respiratory capacity, non-mitochondrial oxygen consumption, and ATP-linked OCR were analyzed through the mito stress test to reflect mitochondrial function. Basal ECAR and Stressed ECAR in response to oligomycin injection were registered, and parameters such as baseline and stressed OCR, baseline and stressed ECAR, and the ratio between baseline OCR/ECAR and the metabolic potential were analyzed through the energy phenotype test. OCR values (pmolO_2_/min) and ECAR values (mpH/min) were normalized to total protein concentration per well (μg/μL) measured by a Bradford assay.

### 4.6. Statistic

All experiments were done at least in triplicate, and data are expressed as mean ± SEM. An unpaired t-test was used for comparisons between the 2 groups. A *p*-value < 0.05 was considered statistically significant. Graph Pad Software, San Diego, CA, was used for all analyses.

## Figures and Tables

**Table 1:** Antibodies and dilutions used for mitochondrial dynamics proteins expression.

	Target Protein/Reactivity	Source/Isotype	Dilution
**Primary antibodies**	OPA1	Rabbit	1:1000
	MFN1	Rabbit	1:1000
	MFN2	Rabbit	1:1000
	TTCII FIS1	Mouse	1:500
	DRP1	Rabbit	1:1000
	Phospho-DRP1 (Ser616)	Rabbit	1:1000
	β—actin	Mouse	1:2000
**Secondary antibodies**	Rabbit	IgG	1:5000
	Mouse	IgG	1:5000

## Data Availability

Data is contained within the article. Additional information is available on request.

## Supplementary Materials

The following supporting information can be downloaded at: https://www.mdpi.com/article/10.3390/ijms24054394/s1.

## References

[1] Giglio R.V., Stoian A.P., Haluzik M., Pafili K., Patti A.M., Rizvi A.A., Ciaccio M., Papanas N., Rizzo M. (2021). Novel molecular markers of cardiovascular disease risk in type 2 diabetes mellitus. Biochim. Biophys. Acta Mol. Basis Dis..

[2] Galicia-Garcia U., Benito-Vicente A., Jebari S., Larrea-Sebal A., Siddiqi H., Uribe K., Ostolaza H., Martín C. (2020). Pathophysiology of Type 2 Diabetes Mellitus. Int. J. Mol. Sci..

[3] Joshi M.S., Williams D., Horlock D., Samarasinghe T., Andrews K.L., Jefferis A.-M., Berger P.J., Chin-Dusting J.P., Kaye D.M. (2015). Role of mitochondrial dysfunction in hyperglycaemia-induced coronary microvascular dysfunction: Protective role of resveratrol. Diabetes Vasc. Dis. Res..

[4] American Diabetes Association (ADA) (2021). 2. Classification and Diagnosis of Diabetes: *Standards of Medical Care in Diabetes—2021*. Diabetes Care.

[5] Grismaldo Rodríguez A., Zamudio Rodríguez J.A., Mendieta C.V., Quijano Gómez S., Sanabria Barrera S., Morales Álvarez L. (2022). Effect of Platelet-Derived Growth Factor C on Mitochondrial Oxidative Stress Induced by High d-Glucose in Human Aortic Endothelial Cells. Pharmaceuticals.

[6] Eelen G., de Zeeuw P., Treps L., Harjes U., Wong B.W., Carmeliet P. (2018). Endothelial Cell Metabolism. Physiol. Rev..

[7] de Zeeuw P., Wong B.W., Carmeliet P. (2015). Metabolic Adaptations in Diabetic Endothelial Cells. Circ. J..

[8] Tenopoulou M., Doulias P.-T. (2020). Endothelial nitric oxide synthase-derived nitric oxide in the regulation of metabolism. F1000Research.

[9] Tang X., Luo Y., Chen H.-Z., Liu D.-P. (2014). Mitochondria, endothelial cell function, and vascular diseases. Front. Physiol..

[10] Kim D., Sankaramoorthy A., Roy S. (2020). Downregulation of Drp1 and Fis1 Inhibits Mitochondrial Fission and Prevents High Glucose-Induced Apoptosis in Retinal Endothelial Cells. Cells.

[11] Chen H., Chomyn A., Chan D.C. (2005). Disruption of Fusion Results in Mitochondrial Heterogeneity and Dysfunction. J. Biol. Chem..

[12] Rovira-Llopis S., Bañuls C., Diaz-Morales N., Hernandez-Mijares A., Rocha M., Victor V.M. (2017). Mitochondrial dynamics in type 2 diabetes: Pathophysiological implications. Redox Biol..

[13] Shenouda S.M., Widlansky M.E., Chen K., Xu G., Holbrook M., Tabit C.E., Hamburg N.M., Frame A.A., Caiano T.L., Kluge M.A. (2011). Altered Mitochondrial Dynamics Contributes to Endothelial Dysfunction in Diabetes Mellitus. Circulation.

[14] Makino A., Scott B.T., Dillmann W.H. (2010). Mitochondrial fragmentation and superoxide anion production in coronary endothelial cells from a mouse model of type 1 diabetes. Diabetologia.

[15] Trudeau K., Molina A.J., Guo W., Roy S. (2010). High Glucose Disrupts Mitochondrial Morphology in Retinal Endothelial Cells: Implications for Diabetic Retinopathy. Am. J. Pathol..

[16] Hill B.G., Benavides G.A., Lancaster J.R., Ballinger S., Dell’Italia L., Zhang J., Darley-Usmar V.M. (2012). Integration of cellular bioenergetics with mitochondrial quality control and autophagy. Biol. Chem..

[17] Kashatus J.A., Nascimento A., Myers L.J., Sher A., Byrne F.L., Hoehn K.L., Counter C.M., Kashatus D.F. (2015). Erk2 Phosphorylation of Drp1 Promotes Mitochondrial Fission and MAPK-Driven Tumor Growth. Mol. Cell.

[18] Chang C.R., Blackstone C. (2007). Cyclic AMP-dependent protein kinase phosphorylation of Drp1 regulates its GTPase activity and mitochondrial morphology. J. Biol. Chem..

[19] Divakaruni A.S., Paradyse A., Ferrick D.A., Murphy A.N., Jastroch M. (2014). Analysis and Interpretation of Microplate-Based Oxygen Consumption and pH Data. Methods Enzymol..

[20] Susan W.S.L., Shi Y. (2021). The glycolytic process in endothelial cells and its implications. Acta Pharmacol. Sin..

[21] Li X., Sun X., Carmeliet P. (2019). Hallmarks of Endothelial Cell Metabolism in Health and Disease. Cell Metab..

[22] Yetkin-Arik B., Vogels I.M.C., Neyazi N., van Duinen V., Houtkooper R.H., van Noorden C.J.F., Klaassen I., Schlingemann R.O. (2019). Endothelial tip cells in vitro are less glycolytic and have a more flexible response to metabolic stress than non-tip cells. Sci. Rep..

[23] Koziel A., Woyda-Ploszczyca A., Kicinska A., Jarmuszkiewicz W. (2012). The influence of high glucose on the aerobic metabolism of endothelial EA.hy926 cells. Pflüg. Arch.-Eur. J. Physiol..

[24] Xie L., Shi F., Li Y., Li W., Yu X., Zhao L., Zhou M., Hu J., Luo X., Tang M. (2020). Drp1-dependent remodeling of mitochondrial morphology triggered by EBV-LMP1 increases cisplatin resistance. Signal Transduct. Target. Ther..

[25] Han Y., Kim B., Cho U., Park I.S., Kim S.I., Dhanasekaran D.N., Tsang B.K., Song Y.S. (2019). Mitochondrial fission causes cisplatin resistance under hypoxic conditions via ROS in ovarian cancer cells. Oncogene.

[26] Zhao Q., Lu D., Wang J., Liu B., Cheng H., Mattson M.P., Cheng A. (2019). Calcium dysregulation mediates mitochondrial and neurite outgrowth abnormalities in SOD2 deficient embryonic cerebral cortical neurons. Cell Death Differ..

[27] Zhang S., Gao Y., Wang J. (2017). Advanced glycation end products influence mitochondrial fusion-fission dynamics through RAGE in human aortic endothelial cells. Int. J. Clin. Exp. Pathol..

[28] Shen S., Wang F., Fernandez A., Hu W. (2020). Role of platelet-derived growth factor in type II diabetes mellitus and its complications. Diabetes Vasc. Dis. Res..

[29] Hong S.-G., Shin J., Choi S.Y., Powers J.C., Meister B.M., Sayoc J., Son J.S., Tierney R., Recchia F.A., Brown M.D. (2022). Flow pattern–dependent mitochondrial dynamics regulates the metabolic profile and inflammatory state of endothelial cells. J. Clin. Investig..

[30] Cai J., Wang J., Huang Y., Wu H., Xia T., Xiao J., Chen X., Li H., Qiu Y., Wang Y. (2016). ERK/Drp1-dependent mitochondrial fission is involved in the MSC-induced drug resistance of T-cell acute lymphoblastic leukemia cells. Cell Death Dis..

[31] Haspula D., Vallejos A., Moore T.M., Tomar N., Dash R.K., Hoffmann B.R. (2019). Influence of a Hyperglycemic Microenvironment on a Diabetic Versus Healthy Rat Vascular Endothelium Reveals Distinguishable Mechanistic and Phenotypic Responses. Front. Physiol..

[32] Clyne A.M. (2021). Endothelial response to glucose: Dysfunction, metabolism, and transport. Biochem. Soc. Trans..

[33] Zeng Y., Pan Q., Wang X., Li N., Lin Y., Man F., Xiao F., Guo L. (2019). Impaired Mitochondrial Fusion and Oxidative Phosphorylation Triggered by High Glucose Is Mediated by Tom22 in Endothelial Cells. Oxidative Med. Cell. Longev..

[34] Gay C.G., Winkles J.A. (1991). The half-lives of platelet-derived growth factor A- and B-chain mRNAS are similar in endothelial cells and unaffected by heparin-binding growth factor-1 or cycloheximide. J. Cell. Physiol..

[35] Stab B.R., Martinez L., Grismaldo A., Lerma A., Gutiérrez M.L., Barrera L.A., Sutachan J.J., Albarracín S.L. (2016). Mitochondrial Functional Changes Characterization in Young and Senescent Human Adipose Derived MSCs. Front. Aging Neurosci..

[36] Chaudhry A., Shi R., Luciani D.S. (2020). A pipeline for multidimensional confocal analysis of mitochondrial morphology, function, and dynamics in pancreatic β-cells. Am. J. Physiol. Metab..

[37] Valente A.J., Maddalena L.A., Robb E.L., Moradi F., Stuart J.A. (2017). A simple ImageJ macro tool for analyzing mitochondrial network morphology in mammalian cell culture. Acta Histochem..

[38] Schneider C.A., Rasband W.S., Eliceiri K.W. (2012). NIH Image to ImageJ: 25 Years of image analysis. Nat. Methods.

