# Supplementary material for: Impact of Acute High Glucose on Mitochondrial Function in a Model of Endothelial Cells: Role of PDGF-C
Source: Int J Mol Sci. 2023 Feb 23;24(5):4394. doi: 10.3390/ijms24054394 (PMC10003065; doi:10.3390/ijms24054394)
Supplement: Supplementary file 1 [file ijms-24-04394-s001.zip › ijms-2205274-supplementary.pdf]

Supporting information for

Impact of acute high glucose on mitochondrial function in a model of endothelial cells. Role of PDGF-C.

Adriana Grismaldo Rodríguez [1*], Jairo Zamudio Rodríguez [1], Alfonso Barreto [2], Sandra Sanabria-Barrera [3], José Iglesias [1] and Ludis Morales [1*]

[1] Experimental and Computational Biochemistry Group, Faculty of Sciences, Nutrition and Biochemistry Department, Pontificia Universidad Javeriana, Bogotá 110231, Colombia

[2] Immunology and Cell Biology Group, Faculty of Sciences, Microbiology Department, Pontificia Universidad Javeriana, Bogotá 110231, Colombia

[3] Bioengineering FCV Research Group, Department of Innovation and Technological development, Fundación Cardiovascular de Colombia, Floridablanca 680004, Colombia

* Correspondence: mgrismaldo@javeriana.edu.co (A.G.R.); ludis.morales@javeriana.edu.co (L.M.Á.); Tel.: +57-3114566976 (A.G.R.); +57-3132107272 (L.M.A.)

Supplementary Figure S1. Triplicate blots for evaluation of A. MF1, B. MFN2 and C. OPA1 expression.

A. MFN1

Replicate 1

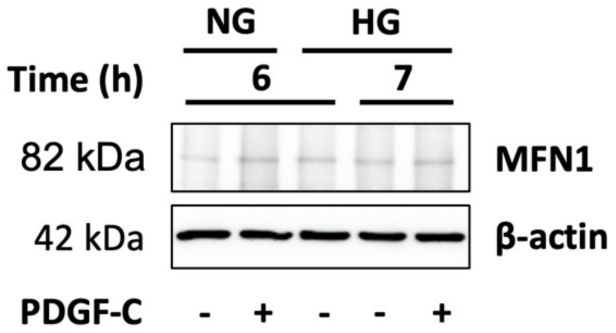

Replicate 2

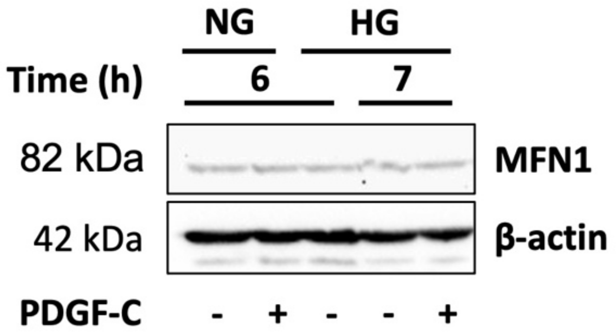

Replicate 3

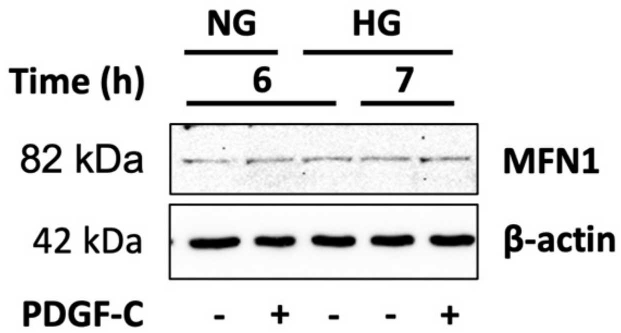

As shown in Figure 2A, replicate 1 was selected as the representative blot for showing in the manuscript.

B. MFN2

Replicate 1

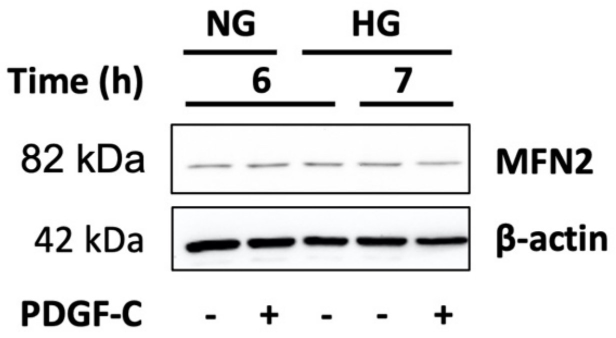

Replicate 2

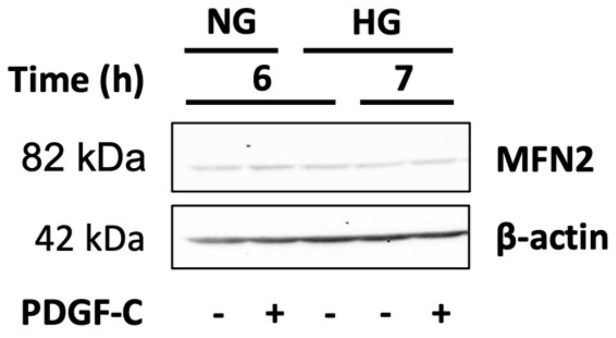

Replicate 3

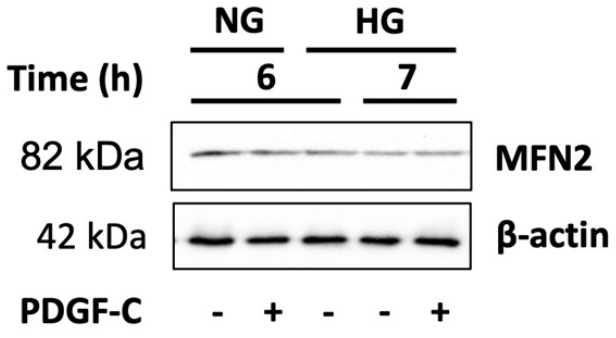

As shown in Figure 2B, replicate 1 was selected as the representative blot for showing in the manuscript.

C. OPA1

Replicate 1

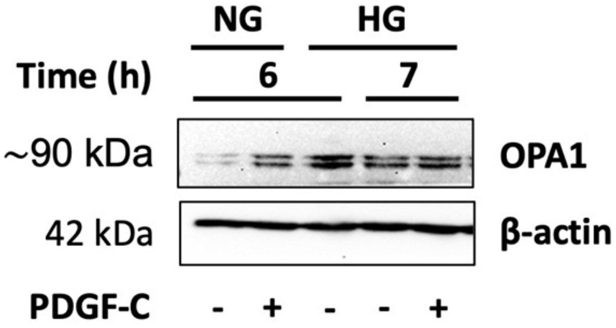

Replicate 2

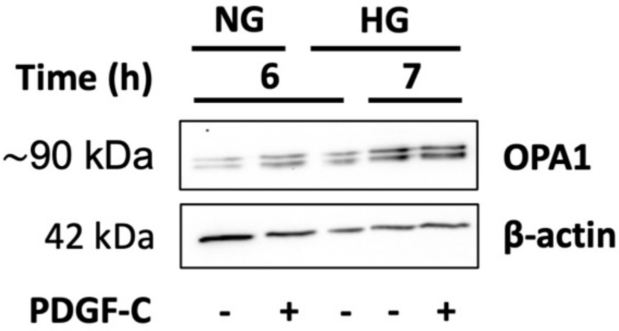

Replicate 3

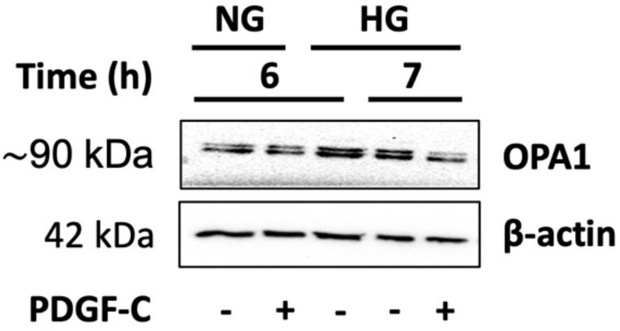

As shown in Figure 2C, replicate 3 was selected as the representative blot for showing in the manuscript.

Supplementary Figure S2. Triplicate blots for evaluation of A. FIS1, B. DRP1 expression and C. DRP^{pSer616} phosphorylation.

A. FIS1

Replicate 1

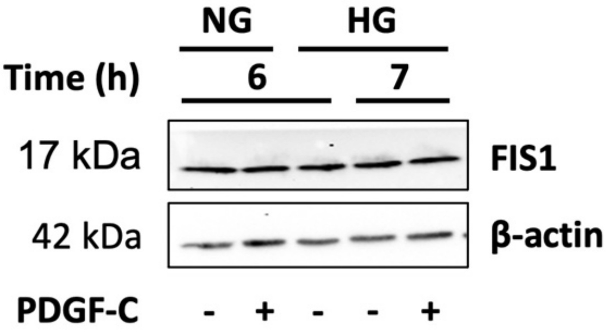

Replicate 2

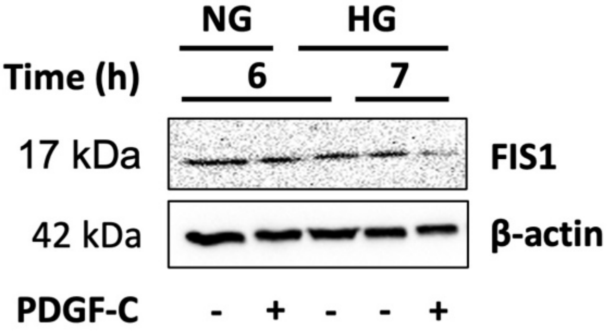

Replicate 3

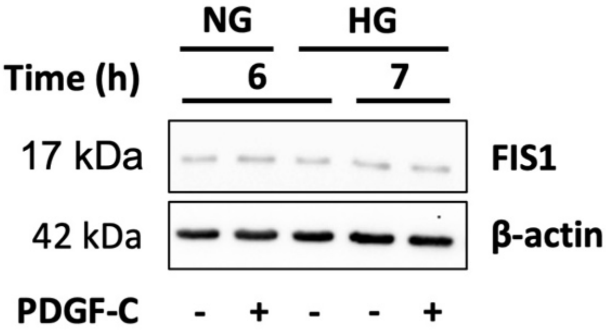

As shown in Figure 3A, replicate 1 was selected as the representative blot for showing in the manuscript.

B. DRP1

Replicate 1

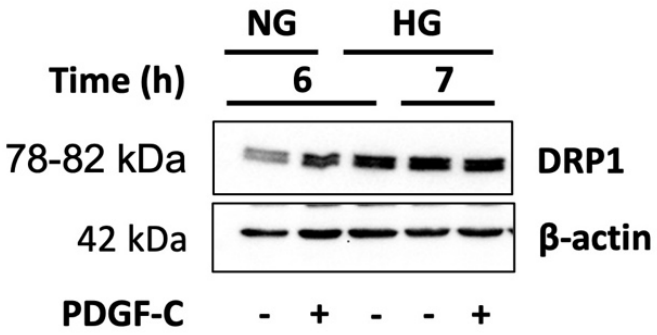

Replicate 2

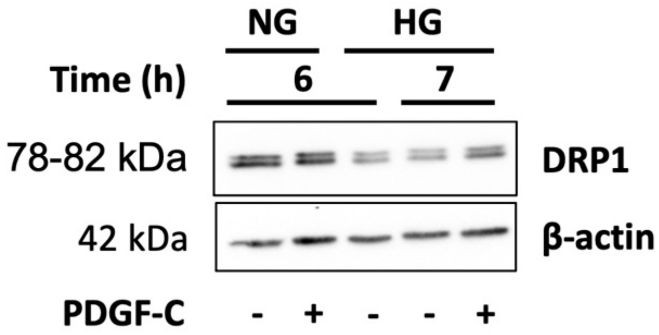

Replicate 3

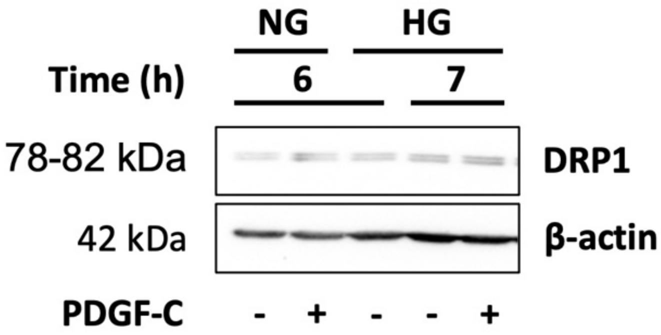

As shown in Figure 3B, replicate 1 was selected as the representative blot for showing in the manuscript.

C. DRP^{pSer616} phosphorylation

Replicate 1

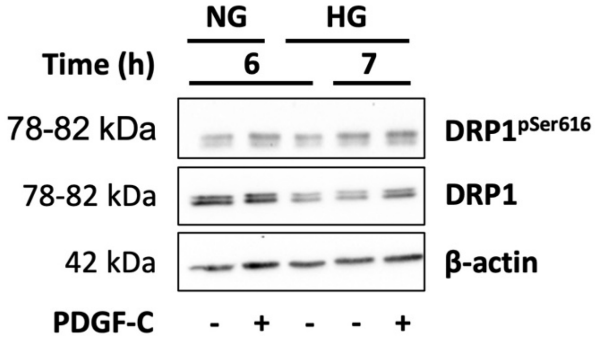

Replicate 2

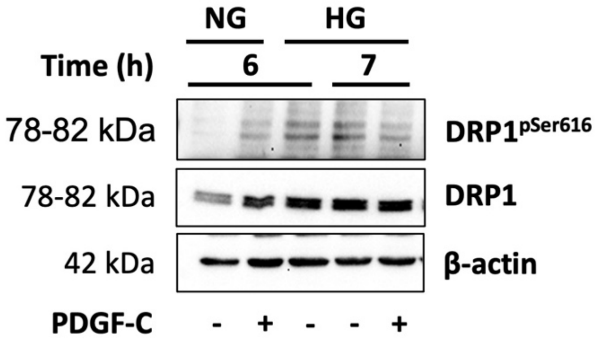

Replicate 3

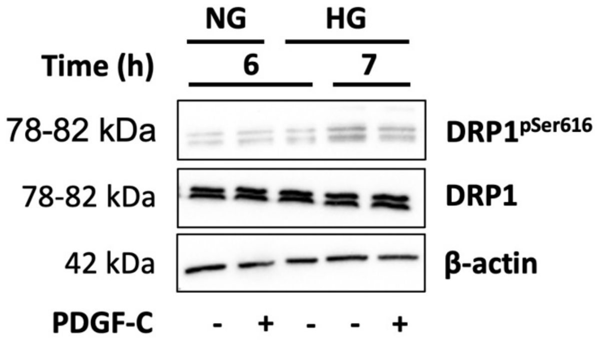

As shown in Figure 3C, replicate 3 was selected as the representative blot for showing in the manuscript.
